# Pathological response following neoadjuvant immunotherapy and imaging characteristics in dMMR/MSI-H locally advanced colorectal cancer

**DOI:** 10.3389/fimmu.2024.1466497

**Published:** 2024-09-27

**Authors:** Zijian Deng, Yajun Luo, Xiaoli Chen, Tao Pan, Yuanyi Rui, Hai Hu, Jin Yan, Ke Zhang, Cheng Luo, Bo Song

**Affiliations:** ^1^ Department of Colorectal Surgery, Sichuan Cancer Hospital & Institute, Sichuan Cancer Center, Cancer Hospital Affiliated to University of Electronic Science and Technology of China, Chengdu, China; ^2^ Department of Imaging Department, Sichuan Cancer Hospital & Institute, Sichuan Cancer Center, Cancer Hospital Affiliated to University of Electronic Science and Technology of China, Chengdu, China; ^3^ Department of Pharmacy, West China Second University Hospital, Sichuan University, Chengdu, China

**Keywords:** colorectal cancer, mismatch repair gene defects, microsatellite highly unstable, PCR, neoadjuvant immunotherapy, PD-1

## Abstract

**Background:**

In recent years, there has been significant research interest in immunotherapy for colorectal cancer (CRC). Specifically, immunotherapy has emerged as the primary treatment for patients with mismatch repair gene defects (dMMR) or microsatellite highly unstable (MSI-H) who have colorectal cancer. Yet, there is currently no data to support the practicality and safety of neoadjuvant immunotherapy for colorectal cancer with dMMR or MSI-H. Therefore, a study was conducted to identify the postoperative pathology, safety profile, and imaging features of patients with dMMR or MSI-H CRC following neoadjuvant immunotherapy.

**Methods:**

The retrospective study was carried out on patients with locally advanced or metastatic CRC who received immunotherapy at Sichuan Cancer Hospital, with approval from the hospital’s ethics committee. The study aimed to assess the short-term effectiveness of immunotherapy by focusing on pathological complete response (pCR) as the primary outcome, while also considering secondary endpoints such as objective response rate, disease-free survival, and safety profile.

**Results:**

Twenty patients with dMMR/MSI-H CRC who underwent neoadjuvant immunotherapy as part of the treatment were enrolled between May 2019 and February 2024 at Sichuan Cancer Hospital. Out of these patients, eight patients received PD-1 blockade monotherapy as neoadjuvant treatment, while 12 were administered a combined therapy of anti-CTLA-4 and anti-PD-1. 12 patients received Nivolumab plus Ipilimumab regimen and 8 patients received PD-1 blockades (2 patients were Pembrolizumab, 2 patients were Sintilimab, 4 patients were Tislelizumab) monotherapy. Additionally, 19 patients underwent surgery after immunotherapy and of these, 15 (75.0%) achieved complete pathological response (pCR), 8 (66.7%) achieved the same on Nivolumab plus Ipilimumab immunotherapy while 7 (87.5%) achieved on PD-1 antibody monotherapy. The overall response rate (ORR) was 75%, with 45.0% of patients experiencing grade I/II immunotherapy-related adverse events. The most frequent adverse event observed was increased ALT i.e. 20%. Notably, no postoperative complications were observed.

**Conclusion:**

Based on the findings, neoadjuvant immunotherapy for colorectal cancer may be both safe and effective in clinical practice. Furthermore, the study suggested that dual immunotherapy could potentially increase the immunotherapy cycle and contribute to a superior pCR rate. However, the conclusion emphasized the need for further prospective clinical trials to validate these results.

## 1 Introduction

Colorectal cancer (CRC) is the third most common form of cancer and the second leading cause of cancer-related deaths worldwide (1). Mismatch repair deficiency (dMMR) occurs in 4–5% of all metastatic colorectal cancers (mCRC) (2, 3). Patients with dMMR/MSI-H CRC have certain characteristics such as poor differentiation, mucinous histology, increased tumor-infiltrating lymphocytes, and a Crohn’s like lymphocytic reaction (4–6). Previous studies have shown that neoadjuvant immunotherapy for CRC is safe and efficacious (7). Neoadjuvant immunotherapy demonstrated promising outcomes in dMMR or MSI-H CRC, especially in rectal cancer patients. Neoadjuvant immunotherapy may lead to a sustained clinical complete response, allowing for organ preservation and avoiding adverse effects on fertility, sexual function, bowel and bladder function after surgery and radiotherapy.

These immunogenic traits make dMMR/MSI-H CRC respond well to treatment with anti-programmed death-1 (PD-1) checkpoint inhibitors. In 2018, PD-1 blockades gained approval for treating metastatic dMMR/MSI-H CRC after standard chemotherapy in the United States (7). The KEYNOTE-016 study showed that dMMR mCRC might benefit from Pembrolizumab (PD-1 inhibitor) monotherapy (5). Subsequently, the CheckMate142 study showed that recurrent dMMR and MSI-H mCRC could benefit from Nivolumab (a PD-1 inhibitor) and Ipilimumab (a CTLA-4 inhibitor) (8). Based on these studies, the Chinese Society of Clinical Oncology guidelines recommend an immune checkpoint inhibitor (ICI) as the second and third-line treatment of dMMR and MSI-H mCRC (9). Following the results of the KEYNOTE-177 study, pembrolizumab was proven to be an effective first-line treatment option in patients with dMMR/MSI-H CRC (10).

Regarding, immunotherapy in perioperative treatment among dMMR CRC, the results of the NICHE 2 study showed high rates of pathological response i.e. 95% (105/111), and complete response 68% (75/111) (11). PD-1 blockade for 6 months alone yields durable recurrence-free responses and provides the potential feasibility for dMMR colon cancer patients to enter a wait-and-watch strategy after neoadjuvant immunotherapy, thereby enabling patients to obtain the benefits of organ function preservation and avoiding the injury and complications caused by surgery (12).

However, data on neoadjuvant immunotherapy for locally advanced or metastatic CRC remained limited. The pCR rate in different clinical trials varied, and whether it was related to the immunotherapy use cycle is still unknown. Here, we presented a study reporting subjects of neoadjuvant immunotherapy for dMMR/MSI-H CRC in our institution. This study was designed to evaluate the clinical features and short-term efficacy of neoadjuvant PD-1 blockade therapy in patients with locally advanced or resectable dMMR/MSI-H CRC. Our study aimed to elucidate the factors contributing to the discrepancy in pCR rate between single-agent and two-drug immunotherapy.

## 2 Methods

### 2.1 Patient selection

The following study was conducted in accordance with the STROBE guidelines (13). It retrospectively included patients with locally advanced or metastatic colorectal cancer (CRC) who received immunotherapy at Sichuan Cancer Hospital. The study was approved by the ethical committee of the Sichuan Cancer Hospital (Ethics Approval Number: SCCHEC-02-2024-069) and informed patient consent for this retrospective analysis was waived. The study enrolled 20 patients with dMMR/MSI-H CRC who underwent neoadjuvant immunotherapy between May 2019 and February 2024 at Sichuan Cancer Hospital. The main inclusion criteria included the pathological diagnosis of CRC with dMMR or MSI-H, clinical stage II~Iva, ECOG performance status of 0 or 1, and patients at least 18 years of age. The exclusion criteria included metastatic lesions that could not be resected prior to radiation therapy, chemotherapy, or surgery for a tumor, and active autoimmune disease requiring systemic treatment or previous treatment with immune checkpoint inhibitors.

### 2.2 Data collection

The clinical features of the patients such as gender, age, family and personal history of malignant tumor, tumor site, degree of differentiation, clinical stage, pathological stage, MMR/MSI status, tumor regression grade (TRG), immunotherapy regimen, adverse events, postoperative complication were collected. All stages were performed following the eighth edition of the American Joint Committee on Cancer (AJCC) (14). Tumor specimen demonstrating mismatch repair deficiency by immunohistochemistry or microsatellite instability as demonstrated by Next Generation Sequence (NGS) or PCR (15).

### 2.3 Outcomes

The primary outcome of the study was pCR, defined as an absence of vital tumor cells in the sampled specimen after resection by pathological examination. Secondary endpoints included the objective response rate by the Response Evaluation Criteria in Solid Tumors RECIST Version 1.1 (RECIST 1.1), disease free survival, and adverse effects as per Common Terminology Criteria for Adverse Events version 5.0 criteria (16).

### 2.4 Immunotherapy regimen

Nivolumab was administered 3mg/kg for 2 cycles and ipilimab 1mg/kg for 1 cycle according to NICHE-2 study protocol. Patients using single-agent immunotherapy received 200mg intravenous infusion every three weeks until the tumor regressed to undergo radical resection. Surgery was performed within 4-6 weeks after the end of medication. PD-1 inhibitors were used in this study including pembrolizumab, sindillizumab and tislelizumab.

### 2.5 Treatment response

The efficacy of neoadjuvant immunotherapy was assessed by RECIST 1.1. Endoscopy and selective biopsy were performed to determine the presence of residual tumor. The pathologic efficacy indexes were ypTNM and TRG scores after immunotherapy. TRG pathological diagnostic criteria for rectal cancer were obtained based on the AJCC system (14). TRG MRI diagnostic criteria for rectal cancer were obtained based on pathological Mandard diagnostic criteria (17).

### 2.6 Statistical analysis

All continuous data was expressed as median with range, presenting other discrete variables as counts and percentages, and using the software program SPSS version 29 (SPSS Inc., Chicago, IL, version 26.0 for Mac) for statistical analysis. Student’s t-test was used to analyze imaging size changes of tumor and lymph nodes before and after treatment. Univariate analyses were performed to analyze the relationship between baseline characteristics and pCR using a logistic regression model. The expected sample size was calculated according to the alternative hypothesis that the PCR with neoadjuvant immunotherapy would be 60% or higher (H1 = 60%) and the null hypothesis that the PCR after nCRT was 25% (H0 = 25%) (18, 19). With α of 5% and power of 90%, 18 cases would be recruited. A total of 20 patients were recruited with dropout incidence of 10%. *P*<0.05 was considered statistically significant.

## 3 Results

### 3.1 Characteristics of the patients

A total of 20 patients with dMMR/MSI-H CRC were included in the study. There was one patient with stage II disease, 16 with stage III, and 3 with stage IV. 85% of the patients had adenocarcinoma and 65% had colon cancer. Among the stage IV patients: one had postoperative recurrence of colon cancer with liver metastasis, where both the primary lesion and metastasis were resected. Another presented with isolated retroperitoneal lymph node metastasis following right hemicolectomy and the third had colon splenic carcinoma with isolated liver metastasis. 20 patients were diagnosed with dMMR by IHC and 12 patients were detected as MSI-H by PCR. Detailed characteristics are outlined in **Table 1**.

**Table 1:** Baseline clinicapathological characteristic of total patients.

Characteristic	NO. (%)
Age (year)	56 (27–71)
Sex	
Male	11 (55)
Female	9 (45)
Tumor site	
Colon	13 (65)
Rectum	5 (25)
Multiple primary colorectal cancer	2 (10)
Histological Grade	
Medium or Well-differentiated	NA
Poor differentiated	6 (30)
Pathological type	
Adenocarcinoma	17 (85)
Mucinous adenocarcinoma	3 (15)
Drug of ICB	
Single-agent	8 (40)
Two-drug	12 (60)
Loss of expression of MMR proteins	
MSH2 only	4 (20)
PMS2 only	2 (10)
MLH1 and PMS2	7 (35)
MSH2 and MSH6	4 (20)
MSH1, MSH2 and PMS2	2 (10)
MSI status	
MSI-H	12 (60)
Not tested	8 (40)
Pathological TNM Stage	
II	1 (5)
III	16 (80)
IVa	3 (15)
Liver only	2
Distant Lymph Node only	1

NA, not avaliable.

All patients underwent immunohistochemical testing for dMMR, and some also underwent MSI gene or NGS testing to confirm MSI-H status. Eight patients received PD-1 blockade monotherapy as neoadjuvant treatment, while 12 patients received a combination of anti-PD-1 and anti-CTLA-4 treatment (**Table 2**). Patients receiving nivolumab plus ipilimumab underwent surgery after 2 treatment cycles, whereas median cycle of single-agent immunotherapy was 5.14 (95%CI, 2.00-8.28). Five patients underwent preoperative chemotherapy, lasting 1-3 cycles, and three patients had received chemotherapy at other hospitals before admission. Additionally, two patients underwent preoperative chemotherapy while awaiting genetic test results. Multiple organ resection was performed in two cases. Notably, Patient 1 had concurrent ascending colon and rectal cancer; pathological and genetic tests showed pMMR and MSS in a patient with elevated colon cancer post-surgery.

**Table 2:** Details of the 20 patients with neoadjuvant ICB therapy.

Patient	Age	Gender	MSI context	RAS/RAF Mutation	Clinic TNM	Drug of ICB	Dose of ICB(mg)	Neoadjuvant Chemotheropy	Surgery
1	56	male	Lynch syndrome, dMMR	NA	cT3N0M0 and cT4aN1M0	Pembrolizumab	200 q3w*3	XELOX+Rectal radiotherapy	Anterior resection + Right hemicolectomy
2	53	female	Lynch syndrome, dMMR, MSI-H	NA	cT4aN2M0	Pembrolizumab	200 q3w*8	NO	Anterior resection + Hysterectomy and double adnexectomy
3	46	male	Sporadic, dMMR,	NA	cT4NxM1	Sintilimab	200 q3w*10	NO	Left hemicolectomy
4	37	male	Sporadic, dMMR,	NA	cT4bN1M1	Sintilimab	200 q3w	NO	Left hemicolectomy
5	50	female	Sporadic, dMMR,	NA	cT3-4aN1M0	Tislelizumab	200 q3w*2	XELOX*1	Right hemicolectomy
6	68	female	Sporadic, dMMR, MSI-H	KRAS	Retroperitoneal lymph node metastasis	Tislelizumab	200 q3w*2	FOLFIRI*1	Retroperitoneal lymphadenectomy
7	48	male	Sporadic, dMMR, MSI-H	NA	cT4bN1M0	Tislelizumab	200 q3w*8	XELOX*3	Radical resection of sigmoid carcinoma + partial cystectomy
8	27	female	Lynch syndrome, dMMR,	NA	cT3N1M0	Tislelizumab	200 q3w*3	NO	Watch and wait
9	71	male	Sporadic, dMMR, MSI-H	KRAS	cT3N1M0	Nivolumab plus ipilimumab	200 + 50	NO	Laparoscopic robot-assisted anterior rectal resection
10	59	male	Lynch syndrome, dMMR, MSI-H	NA	cT4aN1M0	Nivolumab plus ipilimumab	240 + 80	NO	Right hemicolectomy
11	35	male	Lynch syndrome, dMMR, MSI-H	NA	cT3N1M0	Nivolumab plus ipilimumab	240 + 50	NO	Right hemicolectomy
12	67	male	Lynch syndrome, dMMR, MSI-H	KRAS	cT4N1M0	Nivolumab plus ipilimumab	200 + 50	NO	Anterior resection
13	50	female	Sporadic, dMMR, MSI-H	NO	cT3N2M0	Nivolumab plus ipilimumab	200 + 50	FOLFOX*1 Before immunotherapy	Laparoscopic left hemicolectomy
14	34	female	Sporadic, dMMR, MSI-H	NA	cT3N1M0	Nivolumab plus ipilimumab	200 + 64	NO	Laparoscopic right hemicolectomy
15	47	male	Sporadic, dMMR, MSI-H	NA	cT4aN1M0	Nivolumab plus ipilimumab	200 + 65	NO	Laparoscopic left hemicolectomy
16	53	male	Sporadic, dMMR, MSI-H	KRAS	cT4aN1M0	Nivolumab plus ipilimumab	240 + 74	NO	Laparoscopic left hemicolectomy
17	57	female	Sporadic, dMMR, MSI-H	NA	cT4aN1M0	Nivolumab plus ipilimumab	150 + 50	NO	Laparoscopic left hemicolectomy
18	42	male	Sporadic, dMMR, MSI-H	NA	cT3N1M0	Nivolumab plus ipilimumab	200 + 50	NO	Laparoscopic anterior rectal resection
19	34	male	Sporadic, dMMR, MSI-H	NA	cT3N1M0	Nivolumab plus ipilimumab	195 + 50	NO	Laparoscopic right hemicolectomy
20	37	female	Sporadic, dMMR, MSI-H	NA	cT4aN1M0	Nivolumab plus ipilimumab	200 + 44	NO	Laparoscopic right hemicolectomy

ICB, Immune Checkpoint Block; pCR, pathological complete response; PR, partial response; TRG, tumor regression grade; MSI, microsatellite instability; dMMR, mismatch repair-deficient. NA, not avaliable.

### 3.2 Efficacy of neoadjuvant immunotherapy

**Figure 1:**
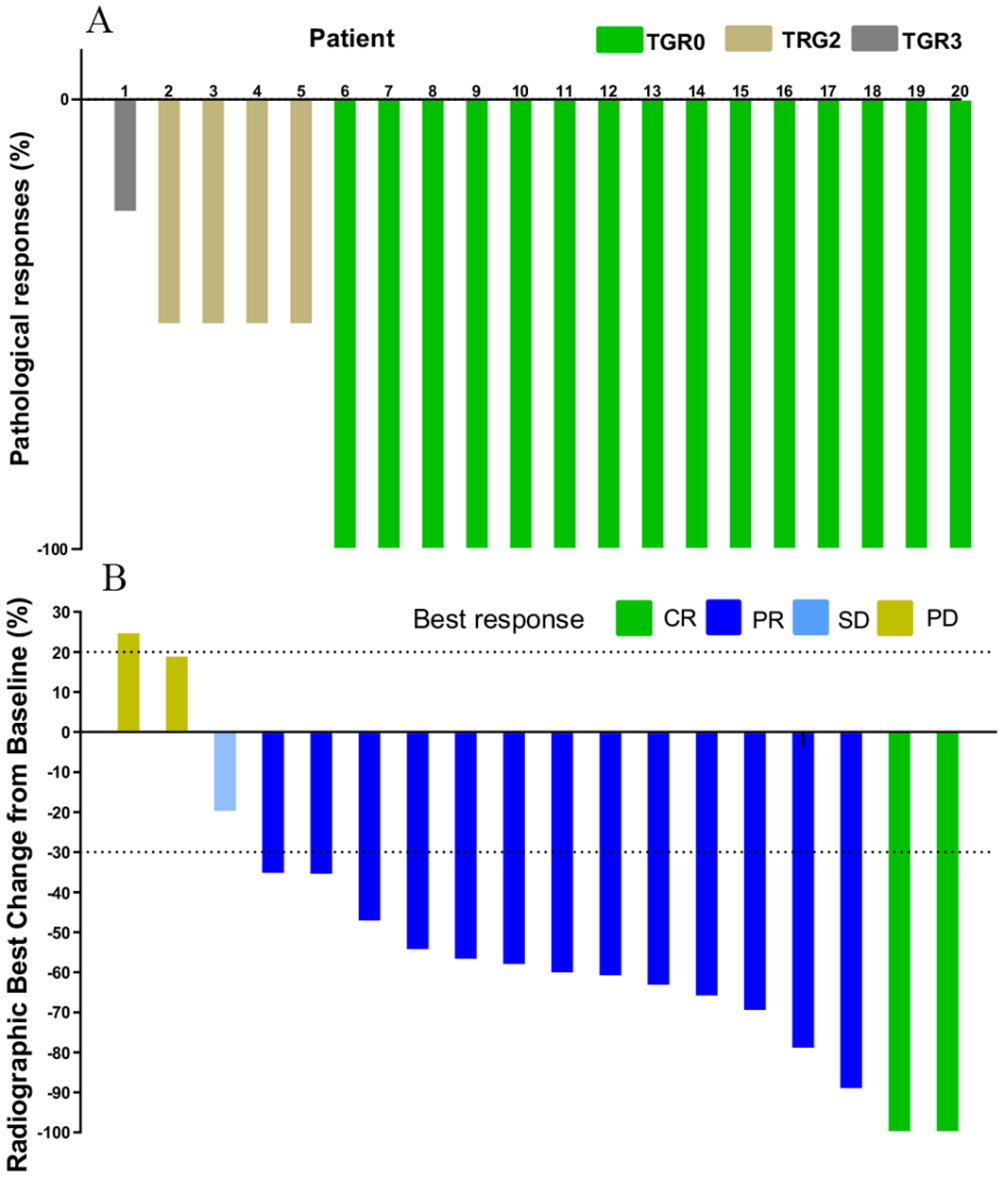
Waterfall plot of efficacy evaluation of neoadjuvant immunotherapy in dMMR/MSI-H CRC. **(A)** Pathological responses(n=20); **(B)** Radiographic responses (n=18).

**Figure 2:**
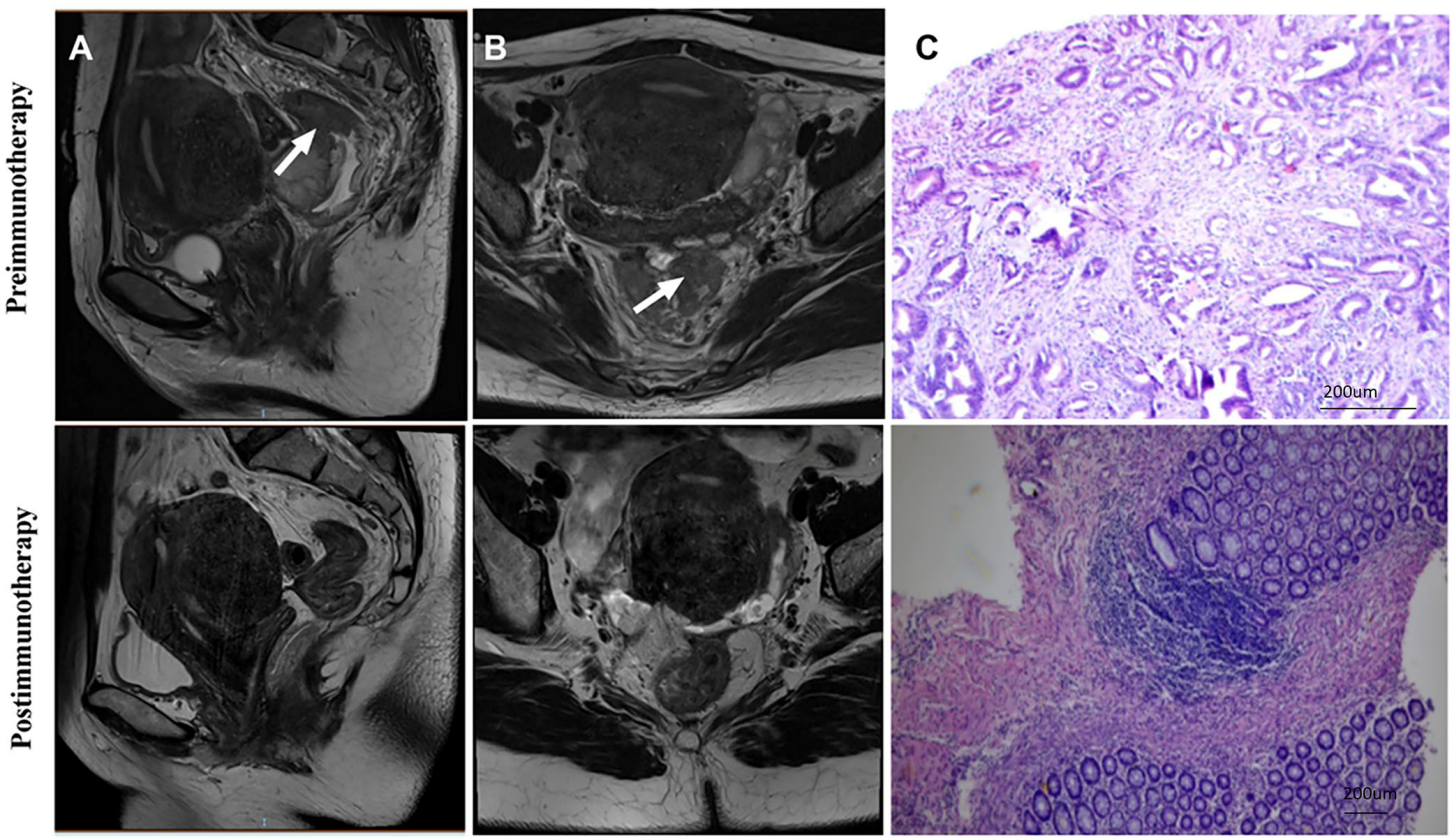
Radiological and pathological responses of 1 pCR patient to monotherapy immunotherapy neoadjuvant therapy (Patient 2 in **Table 2**). **(A)** Sagittal MR View of the pelvis: before immunotherapy VS after immunotherapy; **(B)** MR View of the axial plane of the pelvis: before immunotherapy VS after immunotherapy; **(C)** Pre-biopsy (HE) VS post-biopsy (HE): pre-immunotherapy vs post-immunotherapy. pCR, pathological complete response; MR, magnetic resonance; HE, hematoxylin-eosin.

**Figure 3:**
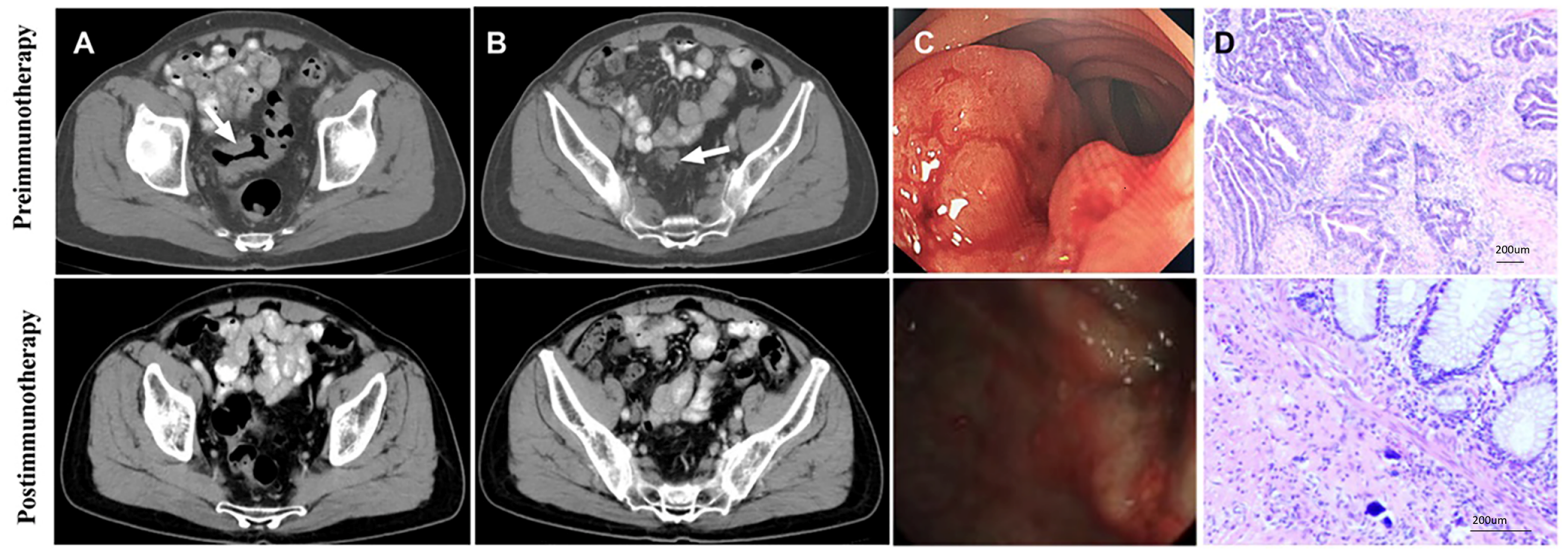
Radiology, colonoscopy, pathological reactions, and postoperative specimens of 1 pCR patient to dual drug immunotherapy and neoadjuvant therapy (Patient 9 in **Table 2**). **(A)** CT view of sagittal tumor: pre-immunotherapy VS post-immunotherapy; **(B)** CT view of lymph nodes around sagittal tumor: before immunotherapy VS after immunotherapy; **(C)** Colonoscopy: pre-immunotherapy VS post-immunotherapy; **(D)** Pre-biopsy (HE) and post-biopsy (HE): pre-immunotherapy VS post-immunotherapy.

Out of the 20 patients enrolled, 19 underwent radical surgery. One patient with anorectal carcinoma achieved imaging PR and opted for observation due to anal retention issues before proceeding with surgical treatment. Among the surgical patients, 15 out of 20 achieved a complete pathological response (**Figure 1A**). Specifically, 7 out of 8 (87.5%) patients who received PD-1 blockades monotherapy achieved a complete pathological response. The radiological and pathological responses of patient 2 in **Table 2** following immunotherapy adjuvant therapy are illustrated in **Figure 2**. Univariate analyses were performed to analyze the relationship between baseline characteristics and pCR by using a logistic regression model, and the results showed no statistical significance (**Table 3**). Additionally, 8 out of 12 (66.7%) patients achieved a complete pathological response with Nivolumab plus Ipilimumab therapy. Patient 9 in **Table 2** attained complete pathological response, and the radiological and pathological response to nivolumab plus ipilimumab adjuvant therapy were depicted in **Figure 3**.

**Table 3:** Univariate analysis of clinical variables for the prediction of pCR.

Characteristic	pCR	Non-pCR	P value
Age (year)			0.157
Sex			
Male	9	3	0.422
Female	6	2	
Tumor site			
Colon	10	3	0.999
Rectum	4	1	
Pathological type			
Adenocarcinoma	13	4	0.998
Mucinous adenocarcinoma	2	1	
TNM Stage			
III	12	5	0.998
IVa	3	0	

### 3.3 Imaging response of tumor after neoadjuvant immunotherapy

**Figure 4:**
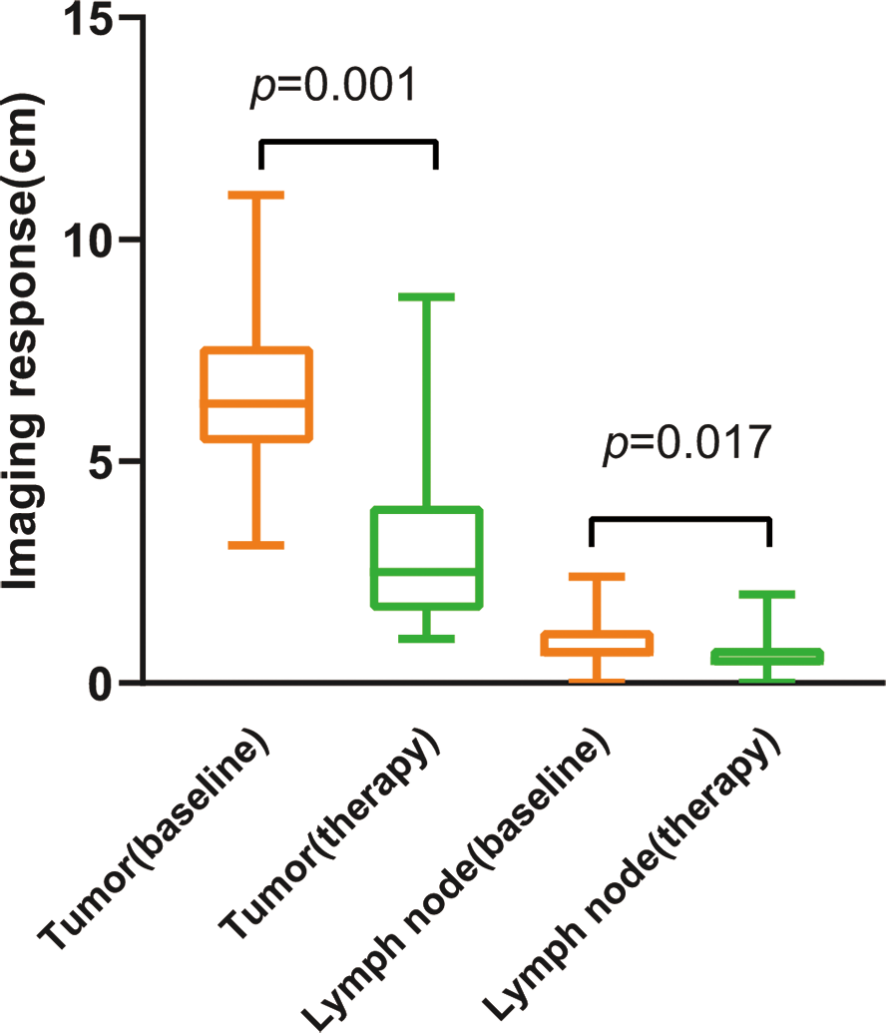
Imaging size changes of tumor and lymph nodes before and after treatment.

**Figure 5:**
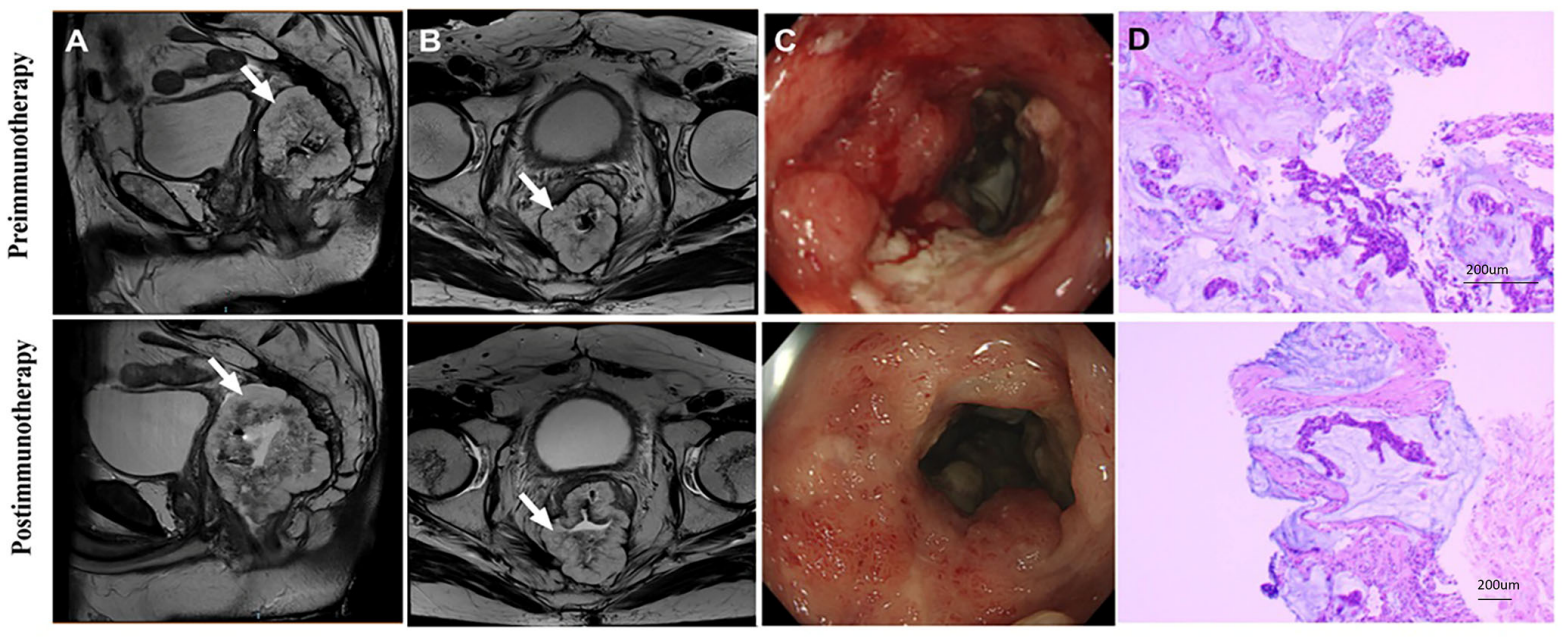
Radiological and pathological reactions of one patient who progressed after receiving dual-drug immunoneoadjuvant therapy (patient 12 in **Table 2**). **(A)** Sagittal MR View of the pelvis: before immunotherapy VS after immunotherapy; **(B)** MR View of the axial plane of the pelvis: before immunotherapy VS after immunotherapy; **(C)** Colonoscopy: pre-immunotherapy VS post-immunotherapy; **(D)**Pre-biopsy (HE) VS post-biopsy (HE): pre-immunotherapy vs post-immunotherapy. MR, magnetic resonance; HE, hematoxylin-eosin.

The changes in imaging for the maximum length diameter and thickness of the primary lesion, as well as the short diameter of the largest lymph node, for the patients before and after treatment are presented in **Table 4** and **Figure 4**. Out of 20 patients, 18 were assessable for the efficacy of neoadjuvant immunotherapy, resulting in an overall response rate (ORR), with 2 complete responses (75%) and 13 partial responses (65%) (**Figure 1B**). Imaging data was unavailable for 2 patients who were examined in other hospitals before treatment. The imaging evaluation was consistent with the pathological evaluation. In addition, one case (Patient 12 in **Table 2**) showed ineffective neoadjuvant immunotherapy, as indicated by tumor progression in a patient with mucinous adenocarcinoma, which was observed in the preoperative MRI. The radiological, colonoscopic, and pathological manifestations of this patient are illustrated in **Figure 5** and were completely consistent with the postoperative pathology.

**Table 4:** Imaging size changes of tumor and lymph nodes before and after treatment.

patient	Tumor maximum diameter before treatment(cm)	Tumor thickness before treatment(cm)	Tumor maximum diameter after treatment(cm)	Tumor thickness after treatment(cm)	Lymph node maximum diameter (short diameter) before treatment(cm)	Lymph node maximum diameter (short diameter) after treatment(cm)	clinical Response	Tumor Response
1	7.2	2.4	1.5	0.5	0	0	1^*^	rectum PCR
2	6.3	2.9	2.5	0.4	2.4	1.2	1*	PCR
3	NA	NA	5.2	2	NA	NA	NA	PCR
4	9.3	3.6	1	0.5	0.8	0.4	PR	PCR
5	4.1	2.2	1.5	0.2	0.7	0.5	PR	PCR
6	2.8	2.4	1.8	1.7	NA	NA	PR	PCR
7	NA	NA	5	0.7	NA	NA	NA	PCR
8	6	1.8	NE	1.2	0.5	0.5	2*	PR
9	3.1	1.2	2	0.7	1.1	0.5	1*	PCR
10	6.7	3.5	2.8	2.1	1	0.4	PR	PCR
11	5	2.3	4	0.8	0.7	0.5	SD	PR
12	7.3	5.5	8.7	4.6	0.6	0.6	PD	PD
13	5.9	1.9	1.5	0.9	0.5	0.4	cCR	PCR
14	5.8	2.3	2.5	0.4	1.3	1.0	PR	PCR
15	7.6	2.9	2.3	2	0.8	0.6	PR	PCR
16	7.7	2.7	3	0.6	0.6	0.4	PR	PCR
17	11	2.1	5	1.5	0.7	0.5	PR	PCR
18	5.9	2.5	2	1.5	0.7	0.5	1*	PCR
19	7.6	3.2	4	1.5	1.3	2	PR	PR
20	4	1.5	5	1.5	1.8	1.3	PD	PD

PR, partial response; SD, stable disease; PD, progressive disease; cCR, clinical complete response; *TRG, tumor regression grade; NA, not available; NE, not evaluable.

### 3.4 Safety and feasibility

Details of adverse events are mentioned in **Table 5**. All adverse events reported spontaneously by the patients or observed by the investigator were recorded during the period of study, with assessments conducted at each treatment cycle, regular follow-up visits, and through patient self-reports. Imaging and laboratory tests were conducted as clinically indicated to identify and grade immunotherapy-related adverse events (irAEs). Among the patients, 45.0% (9/20) experienced grade 1-2 irAEs. The most frequent irAE was ALT increased (20%). Among patients receiving PD-1 blockade monotherapy, 1 out of 8 (12.5%) experienced immune-related adverse reactions, while this rate was 8 out of 12 (66.6%) for patients receiving anti-PD-1 + anti-CTLA-4 immunotherapy. No perioperative deaths were reported, and no postoperative complications were found.

**Table 5:** Adverse events.

Adverse Events	Grade 1-2 (%)	Grade 3-4 (%)
Any	9 (45)	1 (5)
ALT increased	4 (20)	1 (5)
Rash	2 (10)	0 (0)
Thyroid dysfunction	3 (15)	0 (0)
autoimmune myocarditis	1 (5)	0 (0)
gastrointestinal reaction	1 (5)	0 (0)
surgery-related	0 (0)	0 (0)
Anastomotic leak	0 (0)	0 (0)
Obstruction/Ileus	0 (0)	0 (0)
Surgical Site infection	0 (0)	0 (0)
Urinary Retention	0 (0)	0 (0)
Chylous Ascites	0 (0)	0 (0)

## 4 Discussion

The study examined colorectal cancer (CRC) patients with dMMR/MSI-H who received preoperative neoadjuvant immunotherapy at a single center through retrospective analysis. Among the 12 patients treated with dual immunotherapy, the rate of pathological complete response (pCR) was 66.7%, which is consistent with findings from the NICHE-2 study and prior research (11, 20, 21). Notably, the pCR rate was significantly high in patients receiving single-agent immunotherapy (87.5%). The adverse events were generally acceptable (Grade 1-2) and predominantly related to thyroid dysfunction. Thus, preoperative neoadjuvant immunotherapy seemed to be a beneficial and promising strategy.

In a recent study, 16 dMMR patients with locally advanced rectal cancer were treated with dostarlimab (a PD-1 inhibitor) monotherapy for six months (22). All twelve patients who completed the entire treatment regimen achieved complete clinical response (cCR) without requiring chemoradiotherapy or surgery, and there was no reported progression or recurrence during 6-25 months of follow-up (22). Another study enrolled 34 patients with dMMR or MSI-H locally advanced CRC, and they were randomized to receive either Toripalimab (a PD-1 inhibitor) monotherapy (17 cases) or triplimumab combined with Celecoxib (a COX-2 inhibitor) (17 cases) (23). The pCR was notably high at 88% in the triplimumab combined with the Celecoxib group and 65% in the triplimumab monotherapy group (23). Our study demonstrated a pCR of 75.0% among 19 patients who underwent surgery. These findings suggested that neoadjuvant immunotherapy plus COX-2 inhibitors might be a promising option for CRC patients, particularly those for whom anus preservation is challenging. Notably, a female patient, aged 27, with anorectal carcinoma is currently undergoing treatment, and the possibility of adding COX-2 inhibitors to neoadjuvant immunotherapy is under consideration pending further discussions by our multidisciplinary team, as 4 patients in our study did not achieve a complete pathological response despite multidisciplinary team deliberations.

Moreover, none of these patients achieved cCR based on imaging evaluations, and two patients showed disease progression according to imaging assessments. This underscores the importance of comprehensive pre-treatment evaluations, especially in genetically confirmed MSI-H patients. For patients with radiographically evident mucinous adenocarcinoma, the likelihood of poor treatment response should be anticipated. In such cases, adding chemotherapy during immunotherapy or expediting surgery might be warranted.

Interestingly, the pCR among CRC patients treated with nivolumab plus ipilimumab was lower compared to single-drug immunotherapy. Patients receiving nivolumab plus ipilimumab underwent surgery after 2 treatment cycles, whereas median cycle of single-agent immunotherapy was 5.14, potentially reflects insufficient treatment duration with nivolumab plus ipilimumab. Our study results aligned with the NICHE-2 study, showing a 66.7% pathological response rate with ipilimumab plus nivolumab (The NICHE-2 study reported 68% (11, 20)). Currently, there is no consensus regarding the optimal neoadjuvant immunotherapy duration.

In this study, 7 out of 8 (87.5%) patients achieved complete pathological response with -PD-1 blockades monotherapy (including two patients receiving Pembrolizumab, two receiving Sintilimab, and four receiving Tislelizumab). Notably, all 7 patients who received monotherapy achieved complete pathological response. As one patient had not undergone surgery yet, the possibility of a complete pathological response cannot be ruled out. Among patients receiving nivolumab plus ipilimumab, surgery was performed after 2 treatment cycles, with the longest treatment duration reaching 10 cycles. Tailoring treatment cycles according to individual patient characteristics might enhance the pCR rate and implementation of a wait-and-watch strategy is deemed acceptable subsequent to neoadjuvant immunotherapy. Neoadjuvant immunotherapy for patients with early-stage dMMR CRC exhibited a high response rate and low recurrence rate in previous studies (24), but randomized phase III trial with a larger sample size and longer follow-up is warranted to observe the duration of response. The lack of CR detected by imaging in our study, compared to previous studies, may be attributed to several factors, including patient heterogeneity, variability in immunotherapy regimens, differences in imaging and response assessment criteria, and the timing of response evaluations. These factors highlight the complexity of assessing response rates in real-world settings and underscore the need for prospective studies to better characterize CR in diverse patient populations.

The findings of the CheckMate 8HW Phase 3 study (NCT04008030), presented at the 2024 American Society of Clinical Oncology Digestive Oncology Symposium, demonstrated a 79% reduction in disease progression or mortality risk following four to six doses of dual-agent immunotherapy (25). For instance, a patient with mucinous adenocarcinoma progressed despite dual immunotherapy following 2 years of pembrolizumab treatment, indicating the need for individualized immunotherapy strategies.

Immunotherapy has shown promising results in clinical practice but requires careful safety monitoring. A particular concern is immune-related adverse events (irAE), whose mechanisms remain unclear and which commonly affect the lungs, skin, endocrine glands and liver. irAE could manifest with delayed onset, even occurring up to a year after treatment cessation (26). The KEYNOTE-177 study reported a 9% irAE incidence, compared to 13% in the conventional chemotherapy group (10, 27). Timely prediction, identification, and management of irAEs are crucial, with guidelines issued by ASCO, ESMO, and NCCN to assist clinicians in irAE management. Although our study observed immune-related adverse reactions in 12.5% of PD-1 monotherapy patients and 6.6% of anti-PD-1 + anti-CTLA-4 immunotherapy patients, further research is needed to fully understand these outcomes.

Our study has limitations due to its retrospective nature and small sample size, which may result in biases. The diversity of immunotherapy regimens among patients complicates our findings’ interpretation. Therefore, caution is necessary when applying these findings to broader populations. We found no specific clinical variable related to the prediction of pCR, possibly due to the total number of events and sample size, affecting the validity of our logistic model (28). Large studies with extended follow-up durations are needed to understand the correlation between pathological responses and survival rates. While our study highlighted the significance of achieving complete or near-complete pathological responses with neoadjuvant immunotherapy, prospective studies are needed to validate the findings. Some dMMR tumors exhibit resistance to immune checkpoint blockade (ICB) due to various mechanisms, such as an immunosuppressive tumor microenvironment and alterations in antigen presentation pathways. Additionally, genetic alterations beyond dMMR, such as mutations in interferon signaling pathways, and activation of intrinsic tumor cell pathways like WNT/β-catenin, can further contribute to immune evasion. Understanding these resistance mechanisms is crucial for developing combination strategies to overcome resistance and improve the therapeutic outcomes of dMMR tumors. Although our study aimed to replicate existing findings in the context of ICB therapy, we also recognize the importance of providing new insights into the immune dynamics during treatment. Although we did not conduct sequencing of tumor biopsies to assess neoantigen immunoediting directly, immunohistochemistry analysis of pre- and post-treatment samples could indicate trends in immune cell infiltration that correlate with treatment response. Future studies in our cohort will include comprehensive genomic and immune profiling techniques, such as neoantigen sequencing, flow cytometry, and spatial transcriptomics, to better characterize the evolution of immune responses during ICB therapy and identify novel mechanisms of resistance.

In summary, neoadjuvant immunotherapy might be safe and efficacious, but individualized treatment approaches are crucial. For patients exhibiting suboptimal treatment responses, prompt identification and modification of treatment plans were imperative. Ongoing research endeavors are expected to further advance the field of neoadjuvant immunotherapy.

## Data Availability

The original contributions presented in the study are included in the article/supplementary material. Further inquiries can be directed to the corresponding authors.
